# Rendezvous-PIERCE technique: establishing a channel through severe calcification in infrainguinal arterial lesions using needle rendezvous

**DOI:** 10.1186/s42155-024-00445-7

**Published:** 2024-03-15

**Authors:** Takuya Haraguchi, Masanaga Tsujimoto, Yoshifumi Kashima, Katsuhiko Sato, Tsutomu Fujita

**Affiliations:** Department of Cardiology, Asia Medical Group, Sapporo Heart Center, Sapporo Cardio Vascular Clinic, North 49, East 16, 8-1, Higashi Ward, Sapporo City, Hokkaido 007-0849 Japan

**Keywords:** Calcified lesion, Chronic total occlusion, Endovascular therapy, Peripheral artery disease

## Abstract

**Background:**

Severe calcification often prevents device passage and balloon expansion in cases of lower extremity artery disease. To address this limitation, we introduced a novel calcium modification technique called Rendezvous-PIERCE (R-PIERCE).

**Methods:**

A needle was inserted in a retrograde manner and advanced to touch the tip of an antegrade guidewire within the lesion. Then, the guidewire was advanced into the lumen of the needle to achieve partial guidewire externalization, also known as needle rendezvous. The needle was then introduced over the externalized guidewire under wire tension and repeatedly rotated and advanced across the lesion to modify calcified intimal plaques. Notably, this technique can be applied in the opposite direction.

**Results:**

Case 1 involved a 68-year-old male with a calcified occlusion of the anterior tibial artery. An antegrade guidewire reached the midpoint of the occlusion; however, microcatheters and balloons could not pass through the proximal calcification. Therefore, R-PIERCE was used to modify uncrossable lesions. An antegrade 2.5-mm balloon crossed and dilated the lesion, achieving hemostasis at the needle insertion site. The antegrade guidewire successfully crossed the entire lesion and was dilated by the 2.5-mm balloon. Final angiography demonstrated successful flow. In Case 2, an 80-year-old male had a calcified femoropopliteal occlusion. An antegrade guidewire was advanced into the distal superficial femoral artery (SFA); however, no device could follow it. R-PIERCE was performed to modify the calcification from the distal to the medial SFA. The antegrade balloon successfully crossed and dilated obstructed lesions. Furthermore, the antegrade guidewire crossed the entire lesion, and the antegrade balloon was dilated. Final angiography revealed a successful flow without complications.

**Conclusions:**

R-PIERCE is useful for modifying complex calcified lesions during the wiring of occlusive lesions.

**Supplementary Information:**

The online version contains supplementary material available at 10.1186/s42155-024-00445-7.

## Background

Peripheral artery calcification is a common complication in patients with lower extremity artery disease. The presence of calcified lesions may lead to procedural failures because they interfere with lumen expansion and obstruct the passage of the interventional device. The inner PIERCE technique, which involves the insertion of a needle through a vessel to modify intimal calcification, has shown promise in cases of below-the-knee artery (BKA) [1]. This method requires a guidewire to cross the entire lesion and be externalized. However, severe calcifications preventing complete guidewire passage render inner PIERCE inapplicable. To address this limitation, we introduced the Rendezvous-PIERCE (R-PIERCE) technique, which provides effective calcium modification without requiring a full guidewire crossing.

## Materials and methods

An 18- or 20-gauge needle (with outer diameters of 1.3 mm and 0.9 mm, respectively) was selected depending on vessel diameter, the effect of calcium modification, and complications such as bleeding. After the intradermal administration of 1% lidocaine for local anesthesia around the puncture site, the selected needle was inserted retrogradely at an angle of ≤ 45° (30° is the best puncture angle; a shallow angle helps prevent vessel injury and guidewire cutting) from the body surface under angiographical guidance (The use of ultrasound is not recommended in this procedure, as acoustic shadows caused by calcification may interfere with the accurate localization of the target guidewire). The needle was then manipulated to advance to the tip of an antegrade guidewire within a lesion. Upon penetrating the anterior wall of the calcified vessel and contacting the antegrade guidewire, the targeted guidewire was advanced into the needle’s lumen, achieving partial guidewire externalization, an approach known as needle rendezvous [2]. The needle was introduced from the puncture site into the lesion lumen over the externalized guidewire with wire tension. Finally, the needle was repeatedly rotated and advanced across the lesion, preventing device passage until it successfully crossed the lesion. If balloon dilatation was performed under expansion, the procedure was repeated to modify the lesion. This technique could also be applied from the opposite approach.

## Results

**Fig. 1:**
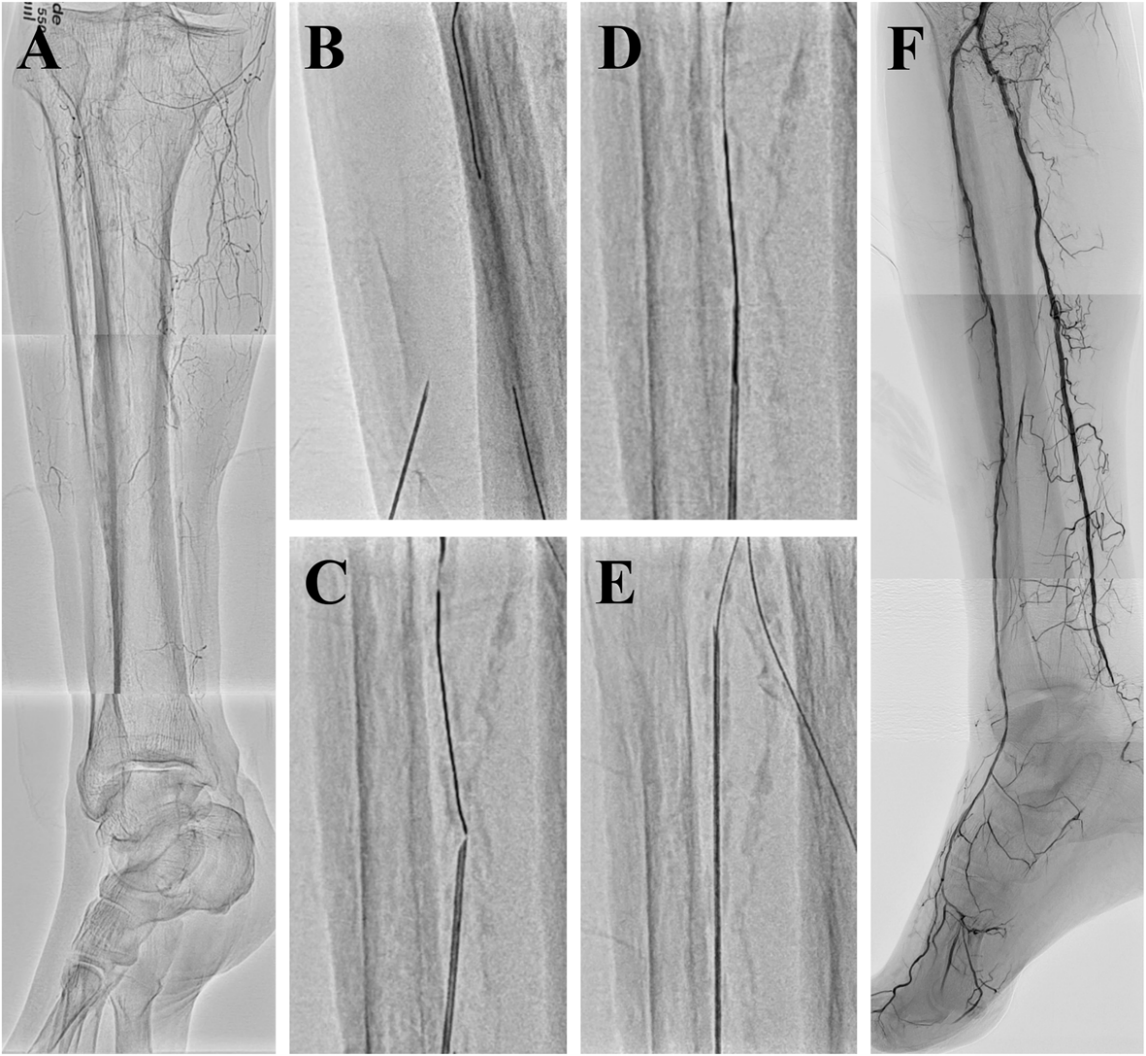
Rendezvous-PIERCE technique for anterior tibial artery calcified occlusion. **A** Initial angiography showing below-the-knee calcified occlusions. **B** Retrograde insertion of a 20-gauge needle toward the antegrade guidewire. **C** Precise alignment of the needle with the tip of the antegrade guidewire. **D** Execution of the needle rendezvous technique. **E** Implementation of the inner PIERCE technique. **F** Post-procedure angiogram revealing successful revascularization without complications

Case 1 involved a 68-year-old male with ischemic rest pain attributed to a BKA occlusion (Fig. 1A). Initial attempts to revascularize the posterior tibial artery occlusion using balloon dilatation were only partially successful due to poor vascular beds, resulting in the persistent rest pain. Consequently, revascularization for the severely calcified occlusion of the anterior tibial artery (ATA) was attempted. An anterior guidewire (Gladius; ASAHI INTECC, Aichi, Japan) successfully reached the middle segment of the occlusion. However, the microcatheters and balloons failed to cross the calcified lesion at the proximal ATA. Although a retrograde approach via the distal ATA was attempted, the retrograde devices failed to pass through the calcification in the middle of the ATA. To address this, R-PIERCE was used to modify calcification in the proximal ATA. Initially, a 20-gauge, 10-cm needle (Medikit, Tokyo, Japan) was inserted retrogradely at a 30° angle and advanced to the tip of the antegrade guidewire (Fig. 1B, C). After needle rendezvous (Fig. 1D), an antegrade 2.5-mm balloon (Oceanus; iVascular, Barcelona, Spain) could not cross the lesion, even during guidewire externalization. Therefore, the 20-gauge needle was retrogradely inserted into the vessel over the externalized guidewire to modify the intimal calcification from the needle insertion site to the proximal lesion (Fig. 1E). The R-PIERCE allowed the antegrade microcatheter (Sergeant; iVascular, Barcelona, Spain) to cross the proximal calcified lesion successfully. The retrograde guidewire was advanced into the antegrade microcatheter for complete externalization. The antegrade 2.5-mm balloon successfully crossed and dilated the entire occlusion, and hemostasis was achieved at the needle insertion site. Final angiography demonstrated successful restoration of blood flow (Fig. 1F). The symptoms improved postoperatively and remained asymptomatic at the 6-month follow-up.

**Fig. 2:**
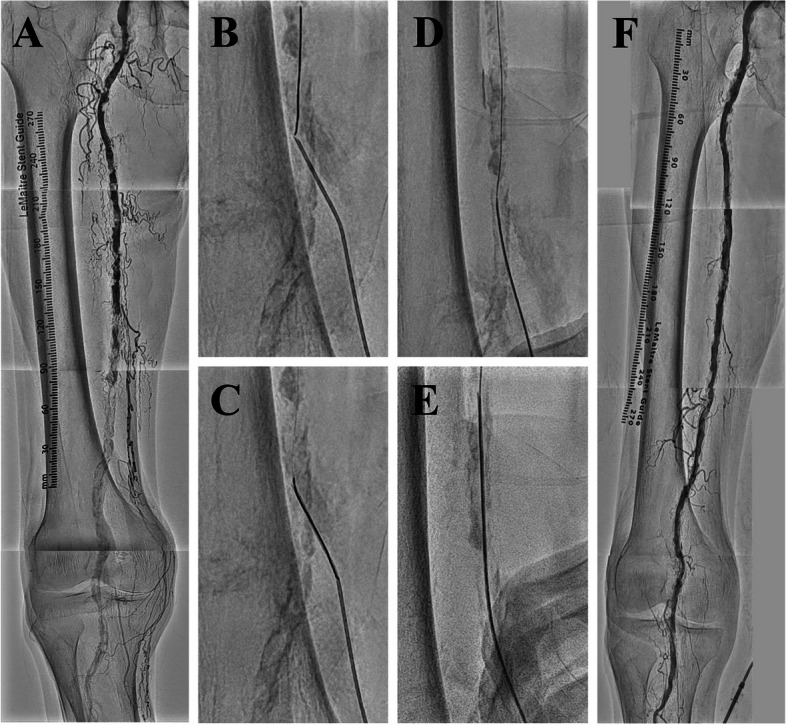
Rendezvous-PIERCE technique for femoropopliteal calcified occlusion. **A** Initial angiography demonstrating femoropopliteal calcified occlusions. **B** Retrograde insertion of a 20-gauge needle targeting the antegrade guidewire. **C** Implementation of the needle rendezvous technique. **D** Introduction of the needle over the externalized guidewire while maintaining the guidewire tension. **E** Performing the inner PIERCE technique. **F** Final angiogram demonstrating successful revascularization

Case 2 involved an 80-year-old male with severe ischemic claudication attributed to a calcified femoropopliteal occlusion (Fig. 2A). After attempting with three penetration guidewires featuring high tip loads to navigate through severely calcified lesions, the anterograde guidewire (Jupiter™ X; Boston Scientific, Marlborough, USA) was successfully advanced into the distal segment of the superficial femoral artery (SFA). However, severe calcification impedes the passage of antegrade devices. Given the extensive complex calcified lesion, retrograde devices may have similar difficulties crossing the calcified lesion as the antegrade approach. Therefore, rather than employing the conventional retrograde approach, the R-PIERCE was implemented. Needle rendezvous using a 20-gauge needle achieved partial guidewire externalization (Fig. 2B, C). Subsequently, the needle was directly inserted through the puncture site to modify the severely calcified lesions (Fig. 2D, E). The R-PIERCE facilitated the successful crossing and dilatation of the antegrade 5.0-mm balloon (JADE; OrbusNeich, Hong Kong, China), which was previously obstructed. The procedure led to a successful crossing and dilation of all lesions with an antegrade balloon. The intervention resulted in satisfactory angiographic outcomes (Fig. 2F). At the 1-year follow-up, the patient remained asymptomatic and required no additional interventions.

These cases are presented in the supplementary materials (Supplementary videos 1 and 2).

## Discussion

To modify calcified lesions using a needle, previous reports have demonstrated the PIERCE technique [3], where a needle is inserted directly into the calcification from outside the body; the inner PIERCE technique [1], where a needle is inserted directly into the calcification from inside the vessel; and the Fracking technique [4], where a needle is inserted into a deep calcification to modify it using hydraulic pressure. The inner PIERCE technique is preferred for BKA lesions due to the small size of the target vessel, which makes the PIERCE or Fracking techniques ineffective. However, the inner PIERCE technique cannot be performed using the previously reported method unless the guidewire passes completely through the lesion while being externalized. The unique ability of the R-PIERCE technique to modify calcified lesions in situations in which the guidewire cannot fully cross the lesion extends its applicability to a broader range of clinical scenarios, including infrainguinal lesions. The practicality of the R-PIERCE is evident in cases that cannot be treated with conventional interventions.

A previous comparative study on BKA interventions with and without atherectomy device use reported that the atherectomy use group had reduced 1-year target lesion revascularization compared to the non-use atherectomy group [5]. In our study, a 20-gauge needle was used to create a channel of approximately 0.9 mm in diameter. The R-PIERCE allows successful balloon passage through the lesion and may enable lesion expansion with a larger lumen area than needleless treatment. Further studies are required to evaluate these clinical outcomes.

However, R-PIERCE primarily targets superficial calcifications and has limitations related to the needle length, puncture angle, and vessel tortuosity. In particular, tortuous calcified vessels are less likely to be successfully straightened by even an externalized guidewire, potentially increasing the risk of vessel injury associated with this technique. Careful case selection and application of techniques are essential for optimal outcomes.

## Conclusions

The R-PIERCE technique is useful for modifying complex calcified lesions during the wiring of occlusive lesions. Future studies should focus on evaluating the technical efficacy and clinical outcomes of this novel technique.

### Supplementary Information

**Additional file 1: Supplementary video 1.** Rendezvous-PIERCE for ATA calcified occlusion.

**Additional file 2: Supplementary video 2.** Rendezvous-PIERCE for SFA calcified occlusion.

## Data Availability

The data are available from the corresponding author upon reasonable request.

## Abbreviations

ATA: Anterior tibial artery
BKA: Below-the-knee artery
PIERCE: Percutaneous direct needle puncture of calcified plaque
R-PIERCE: Rendezvous-PIERCE
SFA: Superficial femoral artery
